# Linking Leaf Gas Exchange to Non-Structural Carbohydrate Allocation to Understand the Early Establishment of Young *Quercus* and *Fraxinus* Species

**DOI:** 10.3390/plants15030434

**Published:** 2026-01-30

**Authors:** Elisa Spennati, Sara Gargiulo, Valentino Casolo, Andrea Alessandroni, Marcello Vitale

**Affiliations:** 1 Department of Environmental Biology, Sapienza University of Rome, 00185 Rome, Italy; elisa.spennati@uniroma1.it (E.S.); alessandroniandrea01@gmail.com (A.A.); marcello.vitale@uniroma1.it (M.V.); 2 Department of Agricultural, Food, Environmental and Animal Sciences, University of Udine, 33100 Udine, Italy; sara.gargiulo@uniud.it

**Keywords:** starch, sugars, restoration, tree establishment, reforestation, stomatal conductance

## Abstract

Forest restoration programs are increasingly adopted to mitigate climate change-driven ecosystem degradation, yet the plant functional strategies underpinning successful tree establishment are not fully understood. We investigated the effect of vapour pressure deficit and soil conditions on the interplay between leaf gas exchange and carbon metabolism in three-year-old saplings of different species characterised by distinct functional strategies, as well as non-structural carbohydrate (NSC) partitioning at plant desiccation. We performed two complementary experiments to evaluate interspecific functional differences between *Fraxinus ornus* L., *Quercus cerris* L., and *Quercus pubescens* Willd. in a Mediterranean restored woodland and to compare them with fully irrigated nursery conspecifics. Stomatal sensitivity to closure was similar between species, whereas higher leaf gas exchange and reduced leaf shedding increased twig sugars, as in the case of *F. ornus*, likely contributing to its better establishment. Irrigation augmented gas exchange rates in potted saplings under moderate evaporative demand but overall did not increase NSCs compared with outplanted conspecifics, possibly because of different carbon demand. Desiccated saplings maintained substantial NSCs, but their reduced pools, especially starch, suggested that they were consumed as a response to drought. Overall, findings indicate that NSC allocation can help define proxies of plant performance in restoration programs.

## 1. Introduction

Recent global policies have emphasised the restoration of damaged forest ecosystems as a key approach to tackling the current climate and biodiversity crises [1]. The need for restoration has arisen due to various threats affecting forests worldwide, including fires, pests, and droughts [2]. Traditionally, restoration practice has been dictated by the re-establishment of historical species assemblages, based on the assumption of a static equilibrium. However, climate change-driven environmental variation has prompted a revision of restoration goals, highlighting the need to consider plant ecophysiology [3,4]. Functional traits, which are measurable characteristics (morphological, physiological, or phenological) at individual or other relevant levels of organisation [5,6], have recently received increasing interest as a quantitative tool in restoration plans [4,7]. Functional traits facilitate the selection of local species with contrasting forms and functions to respond to prevailing environmental forcing [8], thereby synergistically advancing biodiversity conservation and ecosystem resilience. Furthermore, traits enable a nuanced quantification of restoration progress, extending beyond traditional community-level metrics, such as plant cover and abundance [4,9].

The coordination of traits defines different functional strategies that contribute to the successful establishment of plants under varying environmental pressures [10]. Among these, drought is a primary limiting factor influencing plant establishment, thereby affecting forest decline and regeneration [11,12]. Tree responses, such as stomatal closure and transpiration declines, occur in response to both soil and atmospheric drought, making it challenging to disentangle their effects under field conditions [13]. One common way to measure atmospheric drought, or atmospheric desiccation strength, is through the vapour pressure deficit (VPD) [14]. While some studies indicated that stomatal conductance is more strongly influenced by VPD than by soil moisture [15], others showed that this sensitivity is modulated by soil water availability [13,16]. Furthermore, stomatal sensitivity to VPD, referred to as the slope between stomatal conductance and the natural logarithm of vapour pressure deficit [17], varies across species [18,19]. Fast-growing species [20], which prioritise rapid resource acquisition at the cost of lower hydraulic resistance, tend to exhibit higher stomatal sensitivity to VPD [21]. Higher stomatal sensitivity is also expected in more isohydric species, as they usually maintain their water potential relatively stable through stomatal regulation [14,22]. However, this remains an open area of research, since the link between plant water status and stomatal conductance is complex, and the ‘isohydricity’ framework can be misleading, depending on its definition and plant–environment interactions [23,24].

By regulating photosynthesis and transpiration, stomatal conductance represents an early response to drought. Decreased transpiration alleviates xylem tension [17,25], thereby reducing the risk of xylem cavitation, which disrupts water flow and can lead to irreversible cellular dehydration [26]. Conversely, reduced photosynthesis, combined with leaf shedding during lingering water stress either as a water regulation strategy or a consequence of hydraulic damage [27,28], results in a prolonged carbon deficit [29,30]. Under carbon assimilation constraints, trees can rely on previously stored non-structural carbohydrates (NSCs) for sustaining their growth, metabolism and recovery from drought by fuelling cellular turgor maintenance and hydraulic integrity via osmoregulation functions [31,32,33], or the synthesis of defensive compounds [30,34]. Although previous empirical evidence has shown that xylem hydraulic failure is a widespread phenomenon among tree species experiencing drought-induced mortality, it can occur in combination with reductions in NSCs [35]. Currently, whole-plant NSC partitioning in desiccated saplings has been rarely assessed, but it can provide interesting insights, as the mechanisms that can affect tree drought vulnerability (e.g., impairment of phloem transport) remain debated due to the wide range of results obtained under different conditions and the lack of direct testing [36]. Furthermore, the interplay between leaf-level functional behaviour and carbon depletion during drought stress remains complex and not yet fully understood [24].

We focused on a reforestation program in a protected Mediterranean site in central Italy as our case study. First, our research aimed to examine the interplay of leaf gas exchange and NSCs in three-year-old outplanted saplings of *Fraxinus ornus* L. (Oleaceae), *Quercus cerris* L. (Fagaceae), and *Quercus pubescens* Willd. (Fagaceae). We chose these species because they differ in growth rates [37,38] and span the isohydry–anisohydry continuum [39,40,41], with oak species generally exhibiting slower growth and a more anisohydric behaviour. To achieve this, we evaluated whether the species showed different leaf gas exchange responses to VPD at the leaf level (VPD_L_) and soil volumetric water content (θ), leaf shedding, and NSC allocation. Furthermore, we evaluated this interplay between leaf gas exchange responses to VPD and NSCs at the intraspecific level by comparing field saplings with nursery conspecifics of the same age and origin, irrigated at pot capacity. Finally, we aimed to explore the NSC partitioning in plants with completely desiccated epigeal tissues compared to non-desiccated conspecifics at the study site. We expected: (1) both θ and VPD to affect leaf gas exchange responses in the field and *F. ornus* to exhibit greater stomatal sensitivity, more acquisitive leaf function, overall higher photosynthesis activity, and NSC reserves; (2) outplanted saplings to exhibit lower leaf water content, and NSC pools than nursery-grown conspecifics, due to diminished leaf gas exchanges during the summer months; (3) desiccated saplings to exhibit lower but not depleted concentrations of NSCs in all their organs.

## 2. Results

### 2.1. Interspecific Variability in Water Use and Carbon Assimilation

**Figure 1:**
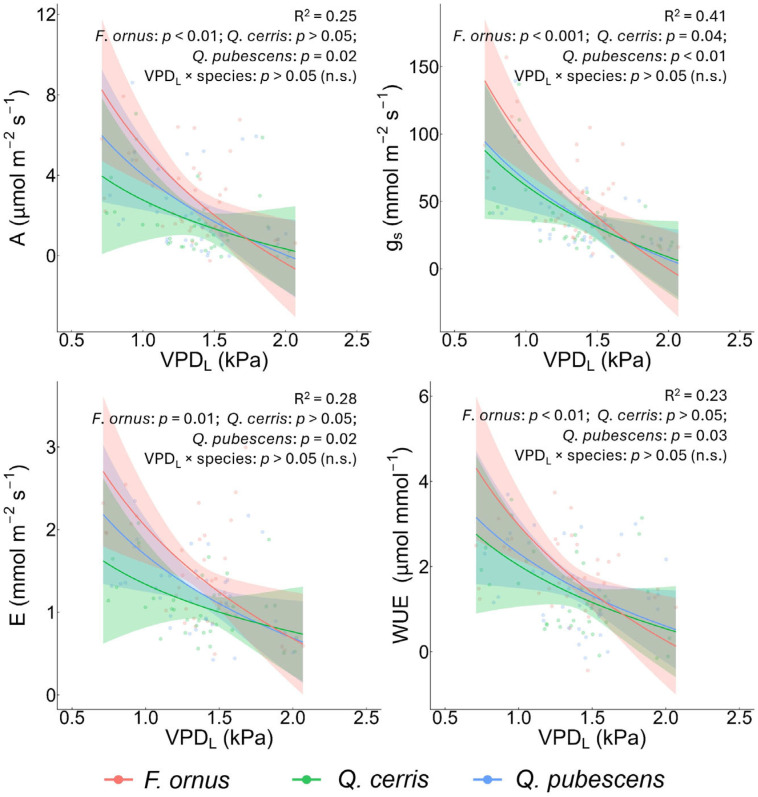
Linear mixed effects models of leaf gas exchange of outplanted saplings as a function of vapour pressure deficit at the leaf level (VPD_L_) during summer and autumn. A: net photosynthesis; g_S_: stomatal conductance; E: transpiration rate; and WUE: water-use efficiency. Points represent mean values measured per plant at each sampling date, while lines show predicted relationships with VPD_L_ with confidence intervals. Colours indicate species: *Fraxinus ornus* L. (red), *Quercus cerris* L. (green), and *Quercus pubescens* Willd. (blue). R^2^ (marginal and conditional) of the models, along with the significance of the VPD_L_ effects on species-specific slopes and of the VPD_L_ × species interaction, are reported at the top of each panel.

Overall, the increase in VPD_L_ significantly reduced net assimilation rate (A, µmol m^−2^ s^−1^), transpiration rate (E, mmol m^−2^ s^−1^), stomatal conductance (g_S_, mmol m^−2^ s^−1^) and water use efficiency (WUE, photosynthesis to transpiration ratio, µmol CO_2_ mmol^−1^ H_2_O) in the studied species, whereas θ had a minor effect (Figure 1 and Figure S1). Specifically, g_S_ declined significantly across varying VPD_L_ in all species (all *p* < 0.05), A decreased significantly in *F. ornus* and *Q. pubescens* (*p* < 0.01; *p* = 0.02, respectively), as did E (*p* = 0.01; *p* = 0.02, respectively), and WUE (*p* < 0.01; *p* = 0.03, respectively). The soil volumetric water content had a significant negative effect only on g_S_ in *F. ornus* (*p* < 0.01), and its interaction with VPD_L_ was significant only in this species for g_S_ and WUE (*p* = 0.04; *p* = 0.01, respectively). Interactions between species and log(VPD_L_), log(θ), or their combination were not significant (Figure 1 and Figure S1). However, interspecific differences emerged for all parameters. Overall, A was higher in *F. ornus* than in *Q. cerris* (*p* = 0.04), as well as E (*p* = 0.02), WUE (*p* = 0.05), and g_S_ (*p* = 0.05). By contrast, no differences were found between the two *Quercus* species and between *F. ornus* and *Q. pubescens* for any of the assessed parameters. The minimum VPD_L_ (≈0.78 kPa) was observed in autumn (September) and the maximum (≈2 kPa) in summer (July), while the minimum θ (≈0.23 m^3^ m^−3^) was observed in autumn (October) and the maximum (≈0.32 m^3^ m^−3^) at the beginning of summer (June). No interspecific differences were found for specific leaf area (SLA), while leaf water content (LWC) was higher in *F. ornus* than in the remaining species (*p* = 0.01) (Figure S2).

**Figure 2:**
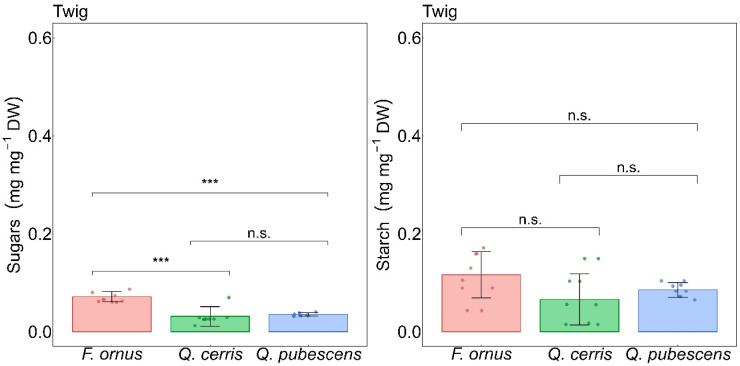
Interspecific differences in non-structural carbohydrates (sugars and starch) in twigs. Statistical significance is indicated as n.s. (not significant), *** (*p* ≤ 0.001). Bars show mean ± standard deviation, with the dots indicating data points and colours indicating species: *Fraxinus ornus* L. (red), *Quercus cerris* L. (green), and *Quercus pubescens* Willd. (blue).

**Figure 3:**
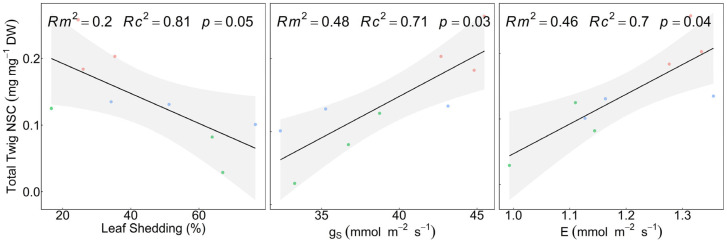
Linear mixed-effect models between leaf gas exchange, leaf shedding, and total twig non-structural carbohydrates. NSC: non-structural carbohydrates, leaf shedding percentage, g_S_: stomatal conductance, and E: transpiration rate. The regression lines are represented as black lines with confidence intervals. Dots indicate data points, with colours denoting species: *Fraxinus ornus* L. (red), *Quercus cerris* L. (green), and *Quercus pubescens* Willd. (blue). Marginal (R^2^m) and conditional (R^2^c) R^2^ values of the models and *p*-value (*p*) are reported at the top of each panel.

The species exhibited differences in sugar concentrations in the twigs (Figure 2), with *F. ornus* presenting higher soluble NSCs than both *Q. cerris* (*p* < 0.001) and *Q. pubescens* (*p* = 0.001), whereas no differences were observed in the other organs or in starch (Figure S3). The total twig NSCs of all species were negatively correlated with the percentage of leaf shedding (marginal R^2^: R^2^m = 0.20, conditional R^2^: R^2^c = 0.81; *p* = 0.05) and positively correlated with g_S_ (R^2^m = 0.48, R^2^c = 0.71; *p* = 0.03) and E (R^2^m = 0.46, R^2^c = 0.70; *p* = 0.04) (Figure 3). In contrast, A did not significantly affect the total twig NSCs (R^2^m = 0.08, R^2^c = 0.68; *p* = 0.29). Plant summer leaf shedding significantly occurred in *Q. cerris* (*p* = 0.05) (Figure S4). The species exhibited contrasting desiccation patterns following the summer season, with *F. ornus* experiencing the lowest percentage (3.33%), whereas *Q. pubescens* showed higher values (37.15%), and *Q. cerris* the highest (42.10%).

### 2.2. Intraspecific Variability in Non-Structural Carbohydrates and Leaf Traits

**Figure 4:**
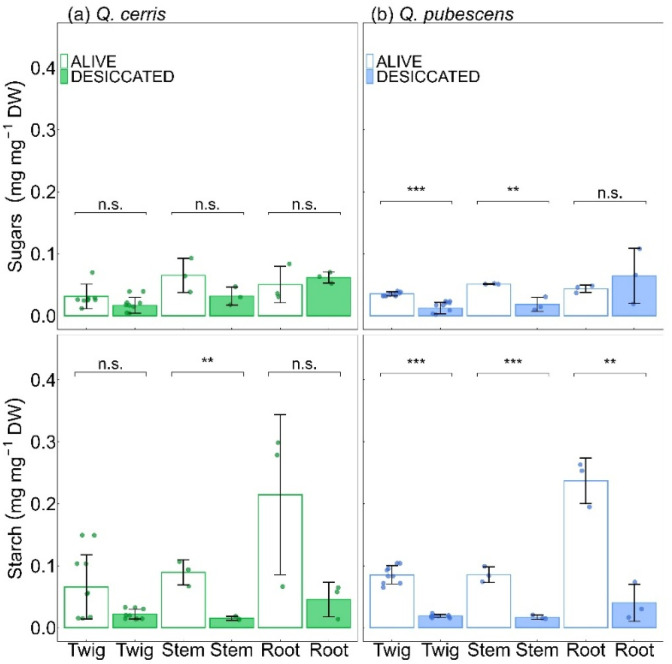
Comparisons of non-structural carbohydrate concentrations (sugars and starch) in desiccated and living non-desiccated conspecific saplings. (**a**) *Quercus cerris* L., (**b**) *Quercus pubescens* Willd. Statistical significance is indicated as n.s. (not significant), ** (*p* ≤ 0.01), and *** (*p* ≤ 0.001). Bars show mean ± standard deviation, with empty bars for living saplings and filled bars for desiccated saplings. Dots indicate data points. Colours indicate species: *Quercus cerris* L. (green), and *Quercus pubescens* Willd. (blue).

NSCs were generally lower in desiccated plants than in living non-desiccated conspecifics, although they were not completely exhausted (Figure 4). Specifically, desiccated saplings showed reduced starch concentration compared to alive conspecifics: in stems of *Q. cerris* (*p* < 0.01) and all woody organs of *Q. pubescens* (*p* < 0.001; *p* = 0.001; *p* < 0.01). Desiccated *Q. pubescens* saplings also showed lower sugar concentrations in twigs (*p* = 0.001) and stems (*p* = 0.01), while *Q. cerris* maintained similar sugar levels in both groups.

**Figure 5:**
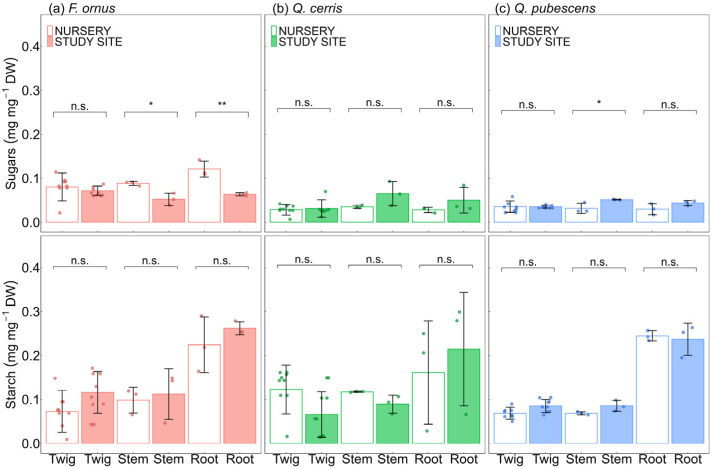
Comparison of non-structural carbohydrate concentrations (sugars and starch) in outplanted and nursery conspecific saplings. (**a**) *Fraxinus ornus* L., (**b**) *Quercus cerris* L., (**c**) *Quercus pubescens* Willd. Statistical significance is indicated as n.s. (not significant), * (*p* ≤ 0.05), and ** (*p* ≤ 0.01). Bars show mean ± standard deviation, with empty bars for nursery saplings irrigated at pot capacity and filled bars for outplanted saplings at the study site. Dots indicate data points and colours indicate species: *Fraxinus ornus* (red), *Quercus cerris* (green), and *Quercus pubescens* (blue).

**Figure 6:**
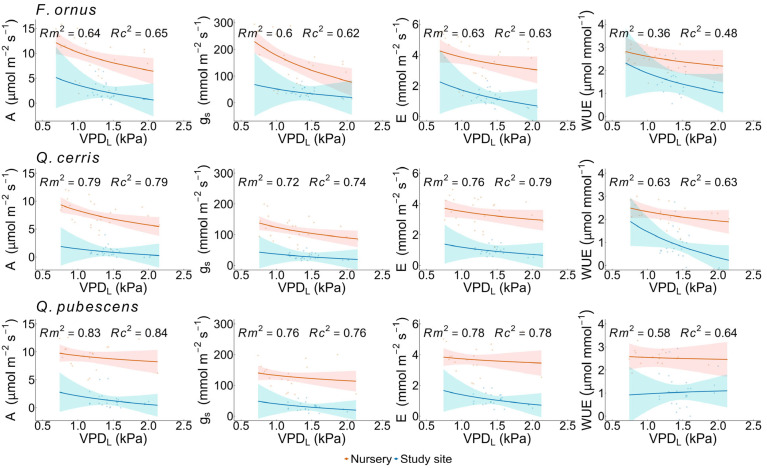
Relationship of leaf gas exchange and vapour pressure deficit at the leaf level (VPD_L_) during the summer season in nursery and outplanted saplings. A: net photosynthesis; g_S_: stomatal conductance; E: transpiration rate; and WUE: water-use efficiency. Points represent mean values per plant and sampling date, while lines indicate the predicted relationships with VPD_L_ with confidence intervals. Colours indicate growing condition: nursery saplings irrigated at pot capacity (orange) and outplanted saplings at the study site under natural conditions (blue). Rows correspond to species, from top to bottom: *Fraxinus ornus* L., *Quercus cerris* L., and *Quercus pubescens* Willd. Marginal (R^2^m) and conditional (R^2^c) R^2^ values of the models are reported at the top of each graph.

Sugars, starch (Figure 5) and mannitol (Figure S5) reserves were similar in outplanted and nursery saplings, with few exceptions. In *Q. pubescens*, nursery saplings had lower (*p* = 0.04) sugar concentration in stems than outplanted saplings. By contrast, in *F. ornus*, sugar concentration was higher in nursery saplings in both stems (*p* = 0.04) and roots (*p* = 0.01). No differences in LWC were found between nursery and outplanted conspecifics throughout the study period (Figure S6). Overall, the nursery saplings were more responsive to summer VPD_L_ than outplanted saplings. In *F. ornus* and *Q. cerris*, both A (all *p* = 0.01) and g_S_ (*p* < 0.001 and *p* = 0.02, respectively) significantly declined with increasing VPD_L_ in the nursery saplings. WUE declined significantly with rising VPD_L_ in outplanted saplings of *Q. cerris* (*p* = 0.04). Interactions between growing condition (nursery vs. study site) and the natural logarithm of VPD_L_ were not significant for any assessed parameter and species (Figure 6). A, g_S_, and E were significantly higher in nursery saplings than in outplanted conspecifics in *F. ornus* (*p* = 0.001 for all parameters), *Q. cerris* (*p* < 0.001 for all parameters), and *Q. pubescens* (*p* < 0.001 for all parameters). WUE was also higher in nursery saplings in *Q. cerris* (*p* = 0.01) and in *Q. pubescens* (*p* = 0.001).

## 3. Discussion

### 3.1. Interspecific Variability in Leaf Gas Exchange and Non-Structural Carbohydrates

Overall, the significant decline of A, g_S_, and E with VPD_L_ indicated that this environmental parameter was a critical driver of leaf gas exchange responses, consistent with previous empirical evidence [17,42,43]. Because the volumetric water content at a depth of 30 cm remained nearly stable and did not fall below critical levels, we could not fully disentangle the effects of soil drought from those of atmospheric drought on leaf gas exchanges under field conditions. This can also explain why soil moisture overall did not significantly affect leaf gas exchanges. However, its significant effect on stomatal conductance in *F. ornus*, but not in *Quercus* species, may reflect differences in their root distribution. Notably, several factors influence stomatal conductance, such as heat stress [44], photosynthetically active radiation [45,46], or intercellular CO_2_ concentration, which fluctuates with mesophyll CO_2_ demand [47], which potentially contributed to the observed patterns besides VPD_L_ and θ.

We expected *F. ornus* to exhibit a sharper decline in stomatal conductance with increasing VPD_L_ and decreasing soil moisture than *Q. cerris* and *Q. pubescens,* due to its relatively more isohydric strategy [39,40,41]. However, we found that all gas exchange parameters showed a similar sensitivity to drought across all species. Thus, interspecific differences in LWC likely did not depend on differences in the slopes of stomatal closure to VPD_L_ and θ across species. The relationship between leaf water potential and stomatal conductance was not explicitly assessed in this study, which prevented us from positioning the species along an isohydry-anisohydry continuum. However, the similar stomatal sensitivity across species may suggest that this response was independent of differences related to the isohydricity framework. While *F. ornus* exhibited a higher net photosynthesis rate and evapotranspiration than *Q. cerris*, its greater water use efficiency indicates that photosynthetic assimilation was less affected by VPD_L_ and θ than water loss in this species. This pattern can be explained by interspecific differences in the relationship between A and g_S_, as E tightly depends on g_S_ (Figure S7). Because the A-g_S_ relationship is saturating [48], moderate declines in g_S_ result in only slight reductions in A, which can lead to higher WUE. When g_S_ declines become more pronounced, A is increasingly limited [48], and the A-g_S_ relationship can be complicated by changes in mesophyll conductance and biochemical capacity [49], which may vary between species. These differences between *F. ornus* and *Q. cerris* likely contributed to the higher twig NSC concentration in the former species, as leaf gas exchange, particularly stomatal conductance and transpiration rate, significantly explained NSC variability in twigs in all species. Conversely, the lack of significant correlation between twig NSCs and net photosynthesis rate may be due to the high temporal variability of this parameter. Furthermore, the pronounced summer leaf shedding observed in *Q. cerris* reduced the amount of photosynthetic tissue, negatively affecting total twig NSCs and further contributing to the observed interspecific differences. Findings of interspecific variability in leaf gas exchanges and twig NSCs align with the generally higher growth rate of *F. ornus* with respect to *Quercus* species [37,38]. While we did not find interspecific differences in SLA, the differences in leaf gas exchange and NSC pools support a more acquisitive strategy in *F. ornus*, especially compared to *Q. cerris*, as expected in our first hypothesis. A more acquisitive strategy can be advantageous for survival during saplings’ early life stages, as saplings may accumulate more reserves to facilitate their recovery from drought [33] or develop deeper roots, thereby favouring the homeostasis of plant water status [50,51]. This functional strategy might have contributed to the better establishment of *F. ornus*, which exhibited lower post-summer desiccation than the *Quercus* species. Consistent with this result, previous evidence has shown high survival rates in the first years of establishment for *F. ornus* saplings [52], even higher than those observed in *Quercus* species, such as *Q. faginea* and *Q. ilex* [53].

### 3.2. Intraspecific Variability in Leaf Gas Exchanges and Non-Structural Carbohydrates Between Nursery and Outplanted Saplings

We found that nursery- and outplanted conspecific plants, which were subjected to similar VPD_L_ ranges but different soil conditions, exhibited different rates of leaf gas exchange during the summer but comparable accumulated NSC pools in autumn. Although leaf gas exchange sensitivity to increasing VPD_L_ appeared independent of the soil conditions, saplings fully irrigated in the nursery maintained higher A, g_S_, and E across the full VPD_L_ range than outplanted saplings under suboptimal soil volumetric water content (θ < θ at field capacity). Notably, although rooting depth was likely similar in potted and outplanted plants because they originated from the same nursery lot, the physical limitation of rooting volume in the nursery may have restricted lateral root development, potentially constraining water uptake and leaf gas exchange rates in potted saplings, independently of the irrigation regime. Overall, we found no clear evidence that nursery saplings had higher LWC and NSC concentrations than outplanted conspecifics, contrary to our second hypothesis. Previous studies found that LWC remained stable under moderate drought stress and declined only under severe drought stress [54,55,56], which can explain the absence of differences observed for this parameter. Several factors could clarify why NSC levels remained stable under varying soil water availability. Within the source–sink framework, carbon balance may be determined by photosynthetic supply regulating sink activities (source control) or by sink demand (e.g., growth, respiration) modulating photosynthesis (sink control), with environmental factors influencing which control predominates [57]. Among the examined parameters, the lower summer photosynthetic activity of outplanted saplings likely limited carbon assimilation in the field. Thus, the overall similar NSC accumulation in nursery and outplanted saplings did not clearly reflect differences in carbon assimilation. This could result from either environmental [58] or carbon supply constraints on growth at the study site, or from contrasting respiration rates, as well as heat stress damage to the photosynthetic system [59] in the two conditions. Alternatively, enhanced leaf gas exchange under lower autumn VPD may have partially compensated for reduced summer assimilation. Similarly, no significant changes in NSCs were observed under imposed drought in previous studies of other woody species [60,61,62]. However, contrasting responses have also been observed, with both decreases [63] and increases [64,65] in NSC levels under water deficit. Moreover, in our study, we observed distinct patterns of NSCs between the nursery and outplanted groups for *F. ornus* and *Q. pubescens*, which may stem from the species-specific variations in carbon supply and demand [66]. Therefore, numerous challenges remain in understanding how different soil moisture levels affect NSC storage and whether increased reserves under these conditions improve the sapling survival after transplanting.

### 3.3. Intraspecific Variability of Non-Structural Carbohydrate Pools Between Living and Desiccated Saplings

Overall, desiccated saplings had lower NSC concentrations, especially starch, than their living non-desiccated counterparts, but their NSC reserves were not completely depleted. This result suggests that desiccated oak saplings still had substantial NSC pools; however, their pools were consumed, likely as a response to drought, in agreement with our third hypothesis. On the one hand, comparable or reduced—but not depleted—sugar levels in desiccated plants indicate that NSCs remained available for sink activities (growth, respiration) or for osmoregulation. On the other hand, the parallel reduction of starch in desiccated tissues is consistent with a sugar-starch conversion, a fairly typical response to dry conditions [62,66,67,68]. In response to water stress, mobilised sugars are indeed used to raise leaf water potential [31], sustain osmoregulation functions to maintain cell turgor [69,70], and repair embolised xylem vessels [71,72,73]. Hydraulic traits were not evaluated in this study, limiting our ability to quantify drought stress in the saplings. Nevertheless, recent evidence suggested that VPD around 2 kPa for several weeks was sufficient to induce substantial embolism in both mesic and xerophilous deciduous tree species, even in the absence of soil drought [74]. At our study site, in addition to the summer VPD_L_ of approximately 2 kPa, summer cumulative precipitation was below 50 mm. Therefore, the environmental conditions and the severe decline in leaf gas exchange with VPD_L_ and θ, down to g_S_ values below 50 mmol m^−2^ s^−1^, may suggest a certain degree of drought stress in the outplanted saplings. Our results of reduced starch in desiccated saplings raise essential questions about whether increased NSCs can sustain sapling survival in plantation trials, as suggested by O’ Brien et al. [31] and Piper et al. [75].

## 4. Materials and Methods

### 4.1. Study Site Characterisation

The study was based on an experimental plot established within a reforestation program conducted at the “Palo Laziale” woodland site, located near the coastline in Italy, approximately 40 km from Rome (41°56′24″ N, 12°06′03″ E) (Figure S8). The woodland is legally protected as it is part of the European Natura 2000 Network (ZSC IT6030022). It extends over 50 hectares, with an altitude ranging from 3 to 10 m above sea level. The soil is classified as clay loam [76], and the climate is characterised as Mediterranean according to the bioclimatic classification applied to Italy by Pesaresi et al. [77]. Throughout the study period (summer-autumn 2023), the mean soil water content (m^3^ H_2_O m^−3^ soil) was nearly constant around 0.25 m^3^ m^−3^ (Figure S9), above the wilting point (θ_W_ = 0.19 m^3^ m^−3^) but below the field capacity (θ_FC_ = 0.32 m^3^ m^−3^) [76]. During summer (June–July) 2023, the mean air temperature and standard deviation were 24.12 ± 2.46 °C, the mean relative humidity and standard deviation were 76.34 ± 6.86%, and the cumulative precipitation was 36.8 mm (Figure S9). In autumn (September–October) 2023, the mean temperature was 21.70 ± 1.74 °C, the relative humidity was 74.69 ± 9.85%, and the cumulative precipitation was 125 mm (Figure S9). The vegetation is predominantly deciduous, consisting mainly of *Quercus* species [78]. The site was affected by a severe oak decline in 2003, primarily due to a severe drought event amplified by the spread of the pathogenic fungus *Biscogniauxia mediterranea* (De Not.) Kuntze (Ascomycota, Xylariales, Graphostromataceae) [79,80]. Thus, the woodland gradually decreased, making way for scrubland and larger clearings [78].

### 4.2. Plant Material and Experimental Design

Three native plant species were selected for the reforestation activities in the Palo Laziale site: *F. ornus*, *Q. cerris*, and *Q. pubescens*. The saplings were cultivated in a forest nursery from locally sourced seeds and transplanted at the study site in 2023 at 3 years old (see Supplementary Methods). The monitored reforested plot extended to 382 m^2^ and comprised 30 *F. ornus*, 38 *Q. cerris*, and 24 *Q. pubescens* positioned in mixed groups with random spacing. The soil volumetric water content was monitored near the experimental plot at 15-min intervals at a depth of 30 cm by means of thermo-hygrometers with data loggers (EL-USB2+, Lascar Electronics, UK). To monitor atmospheric attributes at our study site, mean temperature (°C), relative humidity (%), and precipitation (mm) were collected from the ‘Ladispoli-Palo Laziale’ (RM30CME) monitoring station, situated within the site.

For each species, six potted plants were randomly selected from the nursery lot. The saplings were cultivated in 2.6 L plastic pots filled with a peat–sand substrate (70:30 *v*/*v*). The saplings were monitored at the Botanic Garden of the University of Rome La Sapienza (Rome, 41°53′32” N, 12°27′51” E) and placed on a mulching panel beneath a transparent, retractable roof. To maintain the saplings under optimal watering conditions, we determined the pot capacity gravimetrically. Water was then applied via an automatic irrigation system every two to three days to maintain the pot weight at its capacity (Table S1). The saplings were periodically and randomly rotated to ensure uniform watering conditions. To monitor atmospheric attributes, daily temperature (°C) and relative humidity (%) were recorded hourly using a USB data logger (Easy Log, EL-USB-2) set near the plants. Throughout the summer, the average daily temperature was 24.80 ± 4.94 °C, and the average daily relative humidity was 65.99 ± 17.69%.

Physiological and morphological analyses were conducted in summer and autumn 2023 on both potted lots of 3-year-old *F. ornus*, *Q. cerris*, and *Q. pubescens*, as well as outplanted saplings of the same species, origin and age, in one experimental plot of the reforestation project at the study site (Figure S10).

### 4.3. Leaf Exchange Measurements

Leaf gas exchange was measured between 10:00 a.m. and 1:00 p.m. twelve times at the study site between summer and autumn, and eight times in the nursery during summer, at intervals of approximately one to two weeks. Measurements were conducted on nine fully expanded, healthy leaves per species (three leaves per plant), sampled from three individuals at both the nursery and the study site. The same individuals were measured throughout the study, except in cases of crown desiccation. Leaf temperature, net assimilation rate (µmol m^−2^ s^−1^), transpiration rate (mmol m^−2^ s^−1^), and stomatal conductance (mmol m^−2^ s^−1^) were measured with an open-system gas analyser (CIRAS-2, PP Systems), while water use efficiency (µmol mmol^−1^) was calculated as the ratio between photosynthesis and transpiration rate. Photosynthetically active radiation, reference CO_2_ concentration, relative humidity, and leaf chamber temperature were set at 1000 µmol m^−2^ s^−1^, 425 ppm, 60% and ambient values, respectively.

### 4.4. Leaf Traits

Specific leaf area (m^2^ kg^−1^) and leaf absolute water content (%) were measured in both the nursery and the study site to assess differences in leaf water status and morphology. Leaf trait measurements were performed in the early morning to maintain consistent environmental conditions and were repeated twice during the summer at monthly intervals. Four leaves per species were harvested from the plant’s upper crown to measure the SLA and LWC, each from a different sapling. Fresh leaves were scanned on the same day of sample collection using a flatbed scanner (Brother MFC-L2700DW, Nagoya, Japan), and the leaf area (LA, m^2^) was determined using ImageJ software (version 1.54e) [81]. The samples were oven-dried at 60 °C for 72 h, and their dry weight (DW, kg) was measured using an analytical balance (Gibertini E154, Novate Milanese, Italy) with a resolution of 0.0001 g. SLA was then calculated as the ratio of LA to DW [82]. LWC was obtained from the same leaves used for SLA, following Garnier et al. [83]: LWC = [(FW − DW)/FW] × 100, where FW is the leaf fresh weight (g), and DW is the leaf dry weight (g). Mean leaf area of four leaves per plant was assessed on ten individuals per species in late spring and early autumn at the study site (Table S2) and then multiplied by leaf number to obtain total leaf area (TLA, m^2^). Leaf shedding (%) was computed as: ((N_I_ − N_F_)/N_I_) × 100, where N_I_ and N_F_ denote the initial and final leaf counts, respectively.

### 4.5. Plant Desiccation Assessment

Plant desiccation was evaluated on the above-ground plant organs at 15-day intervals in the plot at the study site from summer to autumn 2023 to compare non-structural carbohydrate pools between completely desiccated and alive, non-desiccated saplings. Saplings were classified as desiccated when both the canopy and phloem were brown and dry, and no buds were present [84]. The saplings assessed comprised 30 *F. ornus*, 38 *Q. cerris*, and 24 *Q. pubescens*, corresponding to the total number of individuals originally outplanted in the plot and alive in late spring.

### 4.6. Non-Structural Carbohydrates

Samples for NSC analyses were collected at 9:00 a.m. in late autumn 2023 to assess starch and sugar concentrations in woody tissues of nursery-grown and outplanted (alive non-desiccated and desiccated) saplings. Twig samples were collected from six saplings per species and group, limiting destructive measurements of stem and root samples to three saplings. From each plant, 2-year-old twigs, stem sections (apex, middle, base; pooled), and coarse roots were collected. We focused on the woody organs, as NSCs are typically accumulated in these organs at the end of the growing season, serving as reserves during winter following leaf drop [85,86]. Comparisons between desiccated and alive non-desiccated conspecifics were restricted to *Q. cerris* and *Q. pubescens*, due to insufficient desiccated *F. ornus* specimens in the monitored plot.

The samples were microwaved at 700 W for 3 min to halt enzymatic activity. After oven-drying at 70 °C for 48 h, samples were ball milled to a fine powder (MM400; Retsch GmbH, Haan, Germany), and 15 ± 1 mg of each dried sample was used for sugar extraction. NSC extraction and analysis followed Landhäusser et al. [87] and Quentin et al. [88], with modifications for small amounts following Gargiulo et al. [89]. Samples were suspended in 80% ethanol, incubated at 80 °C for 30 min, and centrifuged at 14,000 rpm for 3 min three times (Mikro 120, Hettich Zentrifugen, Tuttlingen, Germany) to separate sugars from starch, and the supernatant obtained was dried at 70 °C overnight. Pellet was resuspended in 10 mM Tris-HCl (pH 6.7), and samples were boiled for 1 h to allow starch gelatinisation. Starch was hydrolysed to glucose in two overnight steps at 70 °C using α-amylase (100 U/sample) dissolved in 10 mM Tris-HCl (pH 6.7) and γ-amylase (25 U/sample) dissolved in 25 mM Na Acetate (pH 4.5).

Sugar concentration was determined using the Anthrone assay [90], by reading sample absorbance at 620 nm (Anthrone peak) using a multi-plate reader (Victor3, PerkinElmer, Boston, MA, USA) and compared with absorbance at known glucose concentrations (mg mg^−1^ DW). Starch analysis was performed with the enzymatic method proposed by Bergmeyer and Bernt [91]. Glucose derived from starch digestion was quantified via NADPH formation using hexokinase (0.2 U/sample) and glucose-6-phosphate dehydrogenase (0.5 U/sample) in a buffered solution containing NAD^+^ (50 mM), NaATP (0.4 M) and MgCl_2_ (2 M) for 5 μL of each sample. Reactions were performed at 32 °C, absorbance was measured at 340 nm, and hydrolysed starch was compared with known amounts of commercial amylose (mg mg^−1^ DW), which followed the same procedure as for the samples. Since mannitol is a polyol widely present in the Oleaceae family, we additionally measured mannitol concentration in *F. ornus* [92]. Mannitol concentration was determined enzymatically following Lunn et al. [93], as modified by Gargiulo et al. (under review), using mannitol dehydrogenase (0.6 U/sample) in a buffer solution with Tris-HCl (50 mM) and NAD^+^ (50 mM). Samples were incubated at 40 °C, and NADH formation was measured at 340 nm and compared with known amounts of mannitol.

### 4.7. Vapour Pressure Deficit Calculation

We chose soil volumetric water content (m^3^ m^−3^) as a metric of soil moisture and the vapour pressure deficit at the leaf surface (VPD_L_, kPa) as a proxy for the desiccating strength of the atmosphere at the plant level throughout the monitored period. VPD_L_ was calculated as the difference between the saturated vapour pressure in the leaf (es) and the actual vapour pressure of the ambient air (ea) [14], using the equations described in Andersson-Sköld et al. [94]. Es was calculated based on the leaf temperature measured by the gas exchange analyser. Since the instrument consistently overestimated daily air temperature compared to the meteorological station, we quantified the temperature difference between the two and applied this correction to the leaf temperature values, as described in Lombardi et al. [76]. We verified that daily air temperature accurately predicted the adjusted leaf temperature [76] (Figure S11).

### 4.8. Statistical Analysis

To assess interspecific differences in leaf gas exchanges at the study site (hypothesis 1), we performed linear mixed effects models using the *lme4* package [95] with each gas exchange parameter as a function of the interaction between the natural logarithm of VPD_L_, θ and species, and sapling identity as a random effect. Intraspecific differences between nursery and outplanted saplings were tested using species-specific linear mixed effects models (hypothesis 2), with each gas exchange parameter as response variable and the interaction between the natural logarithm of VPD_L_ and growing condition (nursery vs. study site) as fixed effects, including only summer data to harmonise observation periods. Linear mixed-effects models were also used to test relationships between mean gas exchange parameters, leaf shedding (%) and total twig NSCs, using all study-site data and including species as a random effect. Leaf shedding was assessed using a two-way ANOVA followed by post hoc tests, with species and season as fixed factors and TLA as the response variable.

NSC interspecific (hypothesis 1) and intraspecific differences (hypothesis 2, 3) were tested using one-way ANOVAs with Tukey post hoc tests, separately by plant organ and NSC fraction. LWC and SLA were compared using one-way repeated-measures ANOVAs implemented in the *afex* [96] and *emmeans* [97] R packages (version 4.3.2). Normality of data and homogeneity of variances were checked before the analyses, using quantile–quantile plots and Levene’s test, respectively. All statistical analyses were performed using R version 4.3.2 [98].

## 5. Conclusions

Our study provided new insights into the interplay between leaf gas exchange and non-structural carbohydrate dynamics across and within species, identifying potentially relevant physiological mechanisms that influence reforestation outcomes. While we did not detect differences in stomatal sensitivity to closure between species, *F. ornus* presented higher twig NSC pools at the end of the growing season than both oak species and maintained overall higher photosynthetic activity and water use efficiency than *Q. cerris*, suggesting a more acquisitive strategy. We proposed that its more acquisitive strategy contributed to its higher survival percentage, although additional experimentation is needed. We found that NSCs, particularly starch, were reduced but not depleted in all woody organs of desiccated oak plants, suggesting a starch-to-sugar conversion and indicating that NSCs were maintained at substantial levels during plant desiccation. Irrigation significantly affected leaf gas exchange rates under moderate evaporative demand, but higher carbon assimilation did not necessarily translate into greater NSC accumulation, likely because of differences in C utilisation for sink activities between nursery and outplanted saplings. Measurements of hydraulic and belowground traits would have enhanced our ability to assess plant water status and should be integrated into future work to strengthen mechanistic understanding of species resistance to drought. Given the growing applications of forest restoration projects, clarifying these NSC-related aspects is an essential direction for future research, providing valuable guidance for developing standards and protocols for the use and choice of saplings in reforestation trials.

## Data Availability

The original contributions presented in this study are included in the article/Supplementary Material. Further inquiries can be directed to the corresponding author.

## Supplementary Materials

The following supporting information can be downloaded at: https://www.mdpi.com/article/10.3390/plants15030434/s1, Figure S1: Relationship between leaf gas exchange of outplanted saplings and volumetric water content (θ) during summer and autumn; Figure S2: Interspecific comparison of specific leaf area and leaf water content at the study site; Figure S3: Interspecific comparison of non-structural carbohydrates (sugars and starch) in stems and roots at the study site; Figure S4: Intraspecific comparison of total leaf area (TLA) between late spring and early autumn at the study site; Figure S5: Mannitol concentrations in outplanted and nursery saplings of *F. ornus*; Figure S6: Intraspecific comparison of leaf water content (LWC) between nursery and outplanted saplings in summer; Figure S7: Relationship between leaf transpiration and stomatal conductance; Figure S8: Location and overview of the study site (Palo Laziale Woodland); Figure S9: Meteorological data at the study site; Figure S10: Experimental design; Figure S11: Relationship between leaf and air temperature at the nursery and study site; Supplementary Methods: Plant material; Table S1: Monitoring of nursery pot capacity; Table S2: Estimation of leaf area from allometric data.
