# Supplementary material for: Linking Leaf Gas Exchange to Non-Structural Carbohydrate Allocation to Understand the Early Establishment of Young Quercus and Fraxinus Species
Source: Plants (Basel). 2026 Jan 30;15(3):434. doi: 10.3390/plants15030434 (PMC12899296; doi:10.3390/plants15030434)
Supplement: Supplementary file 1 [file plants-15-00434-s001.zip › plants-4098478-supplementary.pdf]

Supplementary Material

Linking leaf gas exchange to non-structural carbohydrate allocation to understand the early establishment of young *Quercus* and *Fraxinus* species

Elisa Spennati[1], Sara Gargiulo[2], Valentino Casolo[2*], Andrea Alessandroni[1] and Marcello Vitale[1]

[1]Department of Environmental Biology, Sapienza University of Rome, 00185- Rome, Italy

[2]Department of Agricultural, Food, Environmental and Animal Sciences, University of Udine, 33100 - Udine, Italy

*Correspondence: valentino.casolo@uniud.it

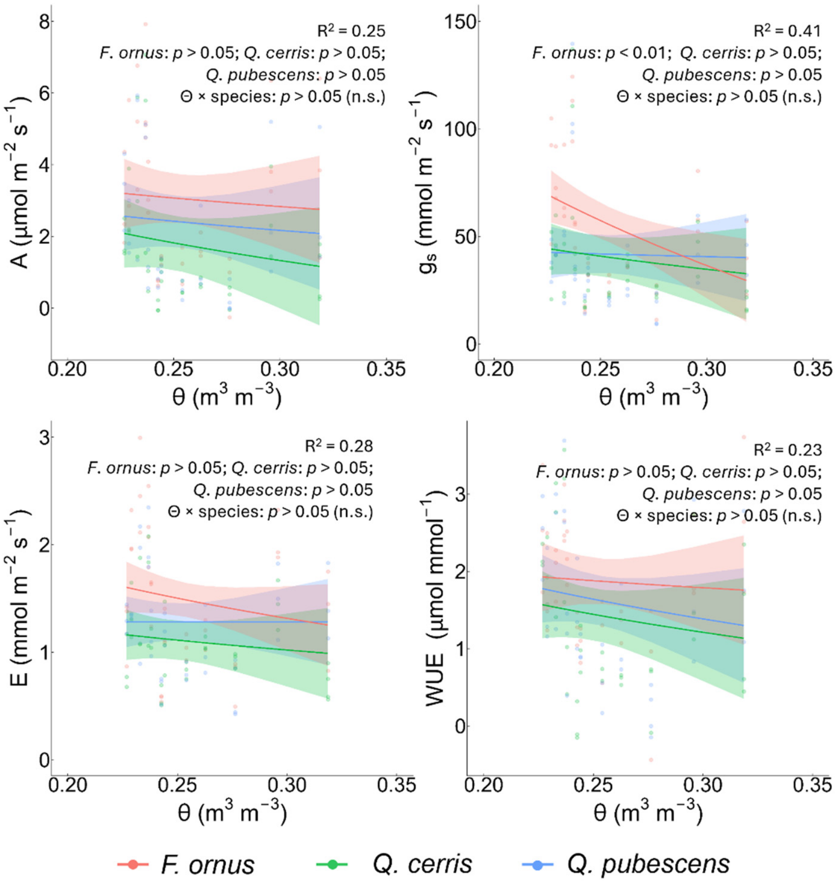

Figure S1. Relationship between leaf gas exchange of outplanted saplings and volumetric water content (θ) during summer and autumn. A: net photosynthesis; g_S: stomatal conductance; E: transpiration rate; WUE: water-use efficiency. Points represent mean values measured per plant at each sampling date, while lines with confidence intervals show predicted relationships with θ. Colours indicate species: *Fraxinus ornus* L. (red), *Quercus cerris* L. (green), and *Quercus pubescens* Willd. (blue). R^2 (marginal and conditional) values of the models, along with the significance of the θ effects on species-specific slopes and of the θ × species interaction, are reported at the top of each panel.

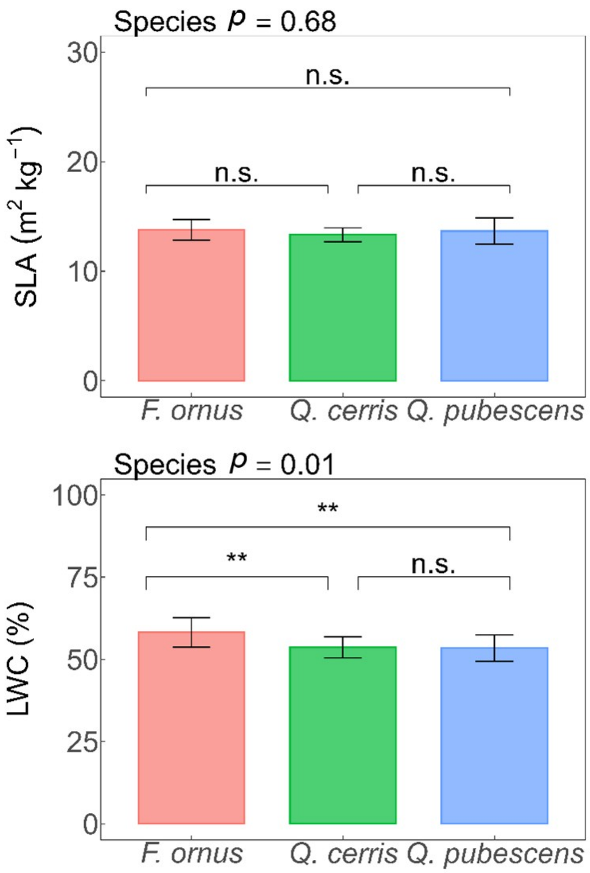

Figure S2. Interspecific comparison of specific leaf area and leaf water content at the study site. SLA: specific leaf area and LWC: leaf water content in outplanted saplings of *Fraxinus ornus* L., *Quercus cerris* L., and *Quercus pubescens* Willd. in summer. Repeated-measures ANOVA results are shown above each panel, while statistical significance is indicated within the panels (n.s. = not significant) and ** ($p \leq 0.01$). Bars represent the mean values per group for the entire period ± standard deviation. Colours indicate species: *F. ornus* (red), *Q. cerris* (green), and *Q. pubescens* (blue).

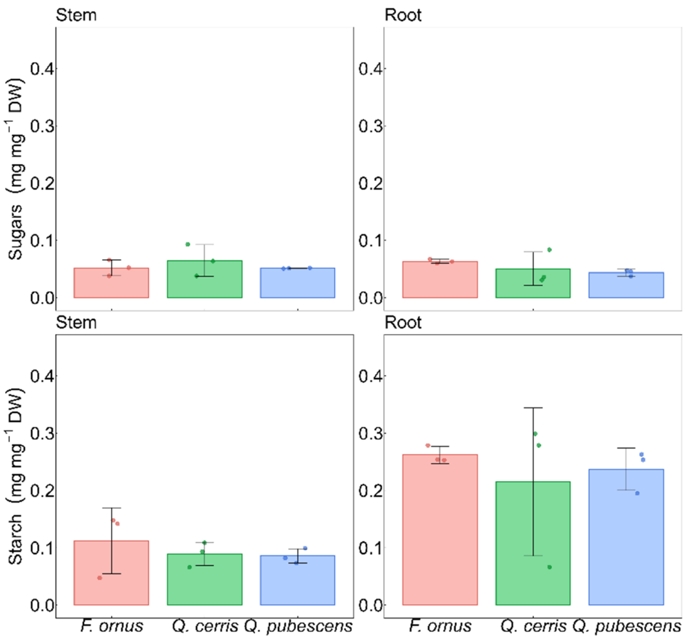

Figure S3. Interspecific comparison of non-structural carbohydrates (sugars and starch) in stems and roots at the study site. Bars show mean ± standard deviation, dots represent the data points, with colours indicating species: *Fraxinus ornus* L. (red), *Quercus cerris* L. (green), and *Quercus pubescens* Willd. (blue). No statistical significance is reported because no interspecific differences were detected.

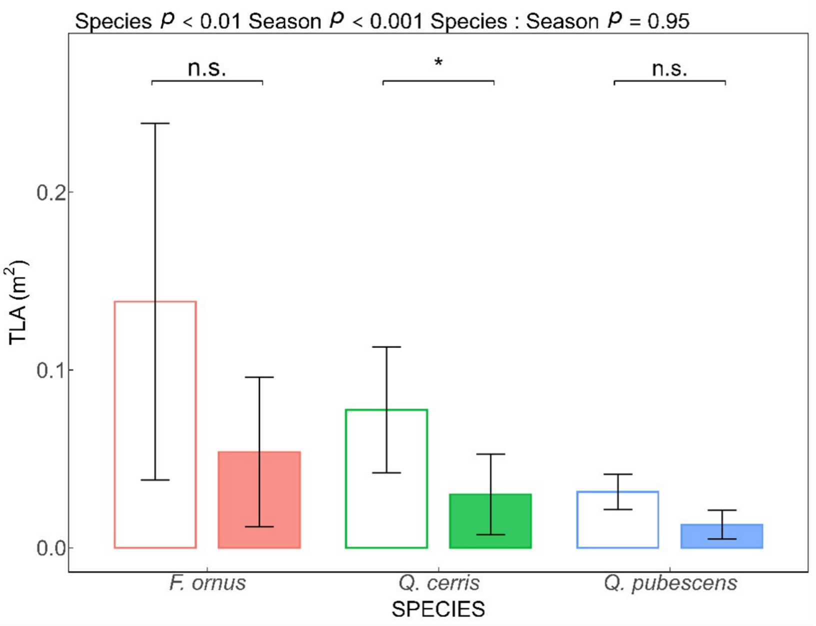

Figure S4. Intraspecific comparison of total leaf area (TLA) between late spring and early autumn at the study site. ANOVA results are shown above the panel. Statistical significance is reported as n.s. (not significant) and * ($p \leq 0.05$). Bars represent mean values per group ± standard deviation, with empty bars for spring and filled bars for autumn. Colours indicate the species: *Fraxinus ornus* L. (red), *Quercus cerris* L. (green), and *Quercus pubescens* Willd. (blue).

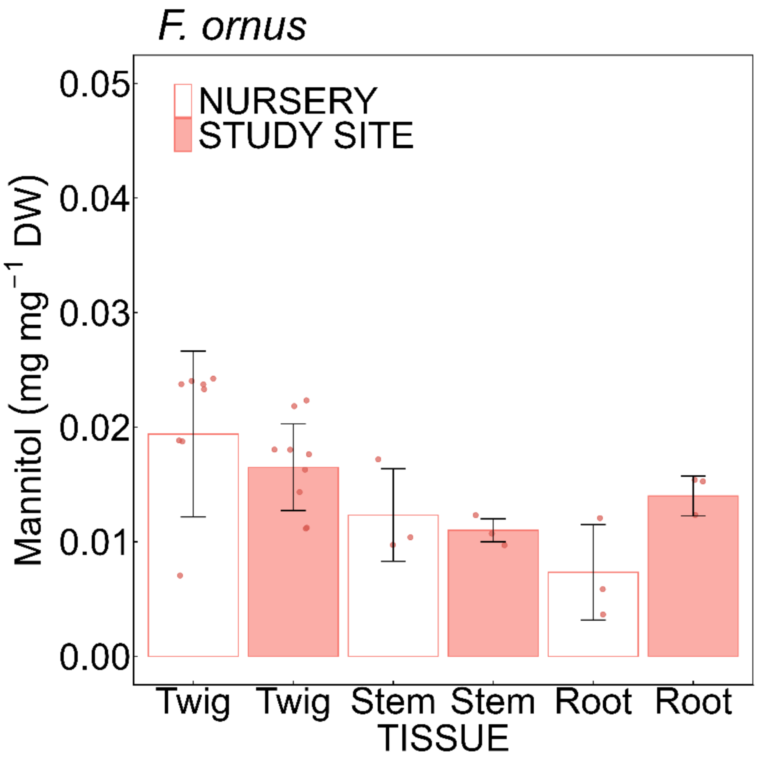

Figure S5. Mannitol concentrations in outplanted and nursery saplings of *Fraxinus ornus* L. No statistical significance is reported because no differences were detected. Bars show mean ± standard deviation, dots represent the data points, with empty bars for nursery saplings and filled bars for outplanted saplings.

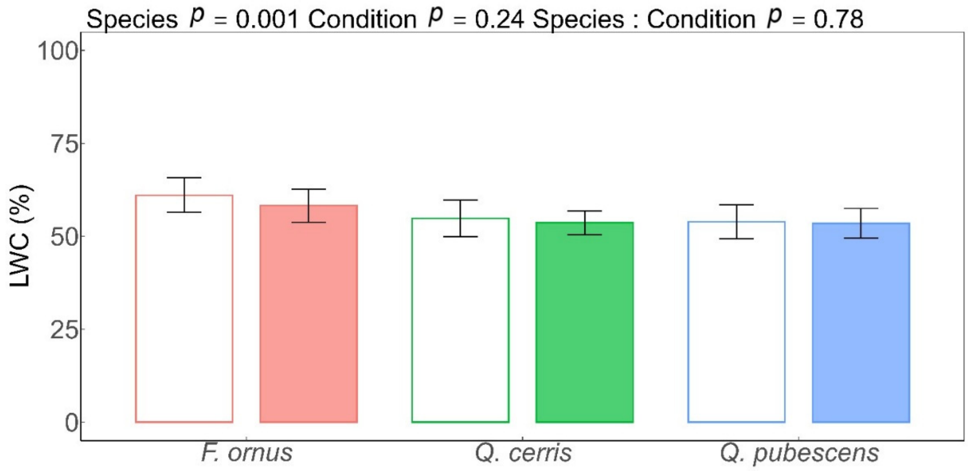

Figure S6. Intraspecific comparison of leaf water content (LWC) between nursery and outplanted saplings in summer. Repeated-measures ANOVA results are shown above the panel. No statistical significance is reported because no differences were detected. Bars represent mean values per group of the entire period ± standard deviation, with empty bars for nursery saplings and filled bars for outplanted saplings. Colours indicate species: *Fraxinus ornus* L. (red), *Quercus cerris* L. (green), and *Quercus pubescens* Willd. (blue).

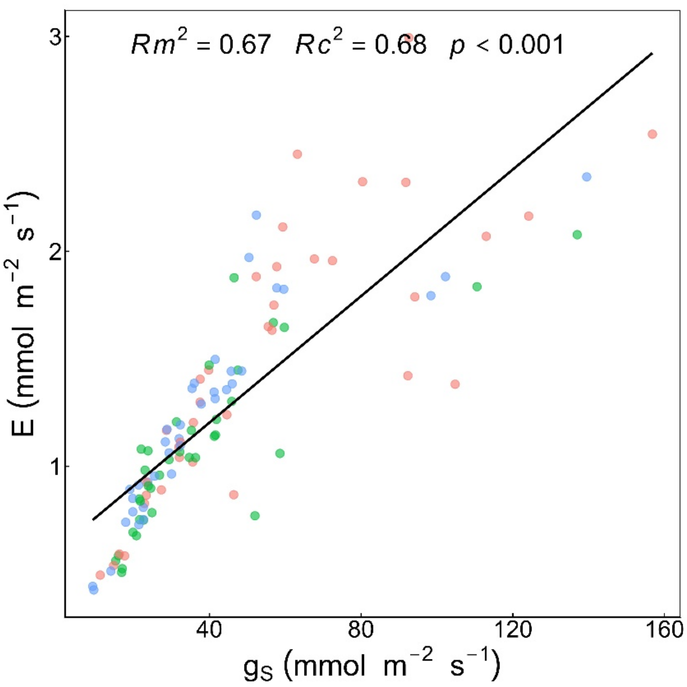

Figure S7. Relationship between leaf transpiration rate and stomatal conductance. Linear mixed-effects model between leaf transpiration rate (E) as a function of stomatal conductance (g_S) in outplanted saplings during summer and autumn, with species included as a random effect. Points represent mean values for each plant and sampling date, with colours indicating species: *Fraxinus ornus* L. (red), *Quercus cerris* L. (green), and *Quercus pubescens* Willd. (blue). Marginal (R^2m) and conditional (R^2c) R^2 values, along with the model *p-value* (*p*), are reported at the top of the panel.

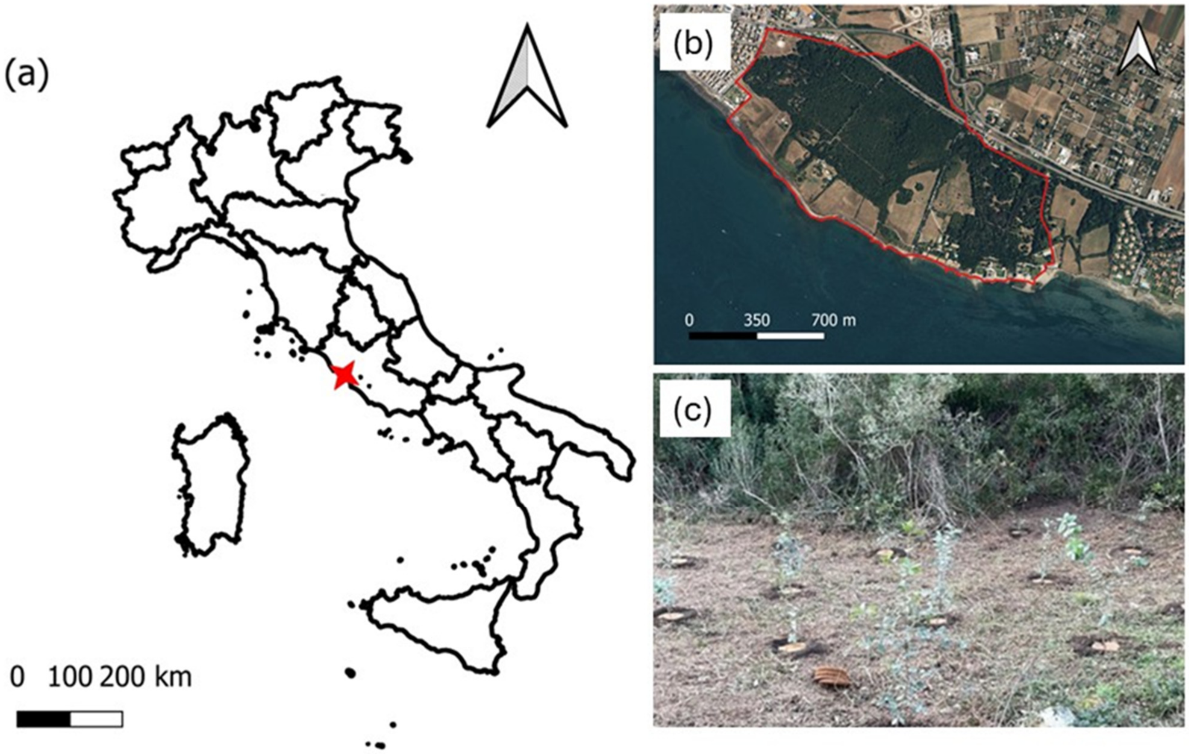

Figure S8. Location and overview of the study site (Palo Laziale Woodland). (a) Map of the Special Area of Conservation (ZSC) Palo Laziale Woodland, indicated by a red symbol (author's image); (b) Aerial view of the study site with ZSC boundaries highlighted with a red line (author's image); (c) Photograph of the reforestation plot within the study site from the technical report of the Life PRIMED project (https://www.lifeprimed.eu/it/). Tree saplings, previously cultivated in the forest nursery, were transplanted at the study site and arranged in mixed groups with random spacing, each protected by a mulching disc at the base of the stem.

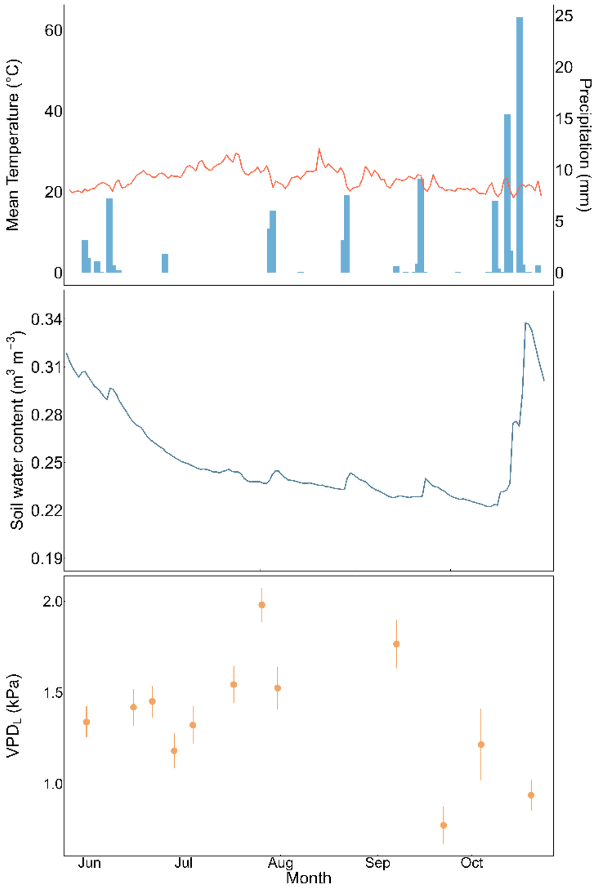

Figure S9. Meteorological data at the study site. Mean daily temperature, precipitation, soil volumetric water content, and vapour pressure deficit at the leaf level (VPD_L) dynamics at the study site from June (summer) to October (Autumn) 2023. Temperature is represented by the red line and precipitation by blue bars (top panel). Soil volumetric water content is shown as a blue line (middle panel). Mean VPD_L with ± standard deviation is displayed as orange points with error bars (bottom panel).

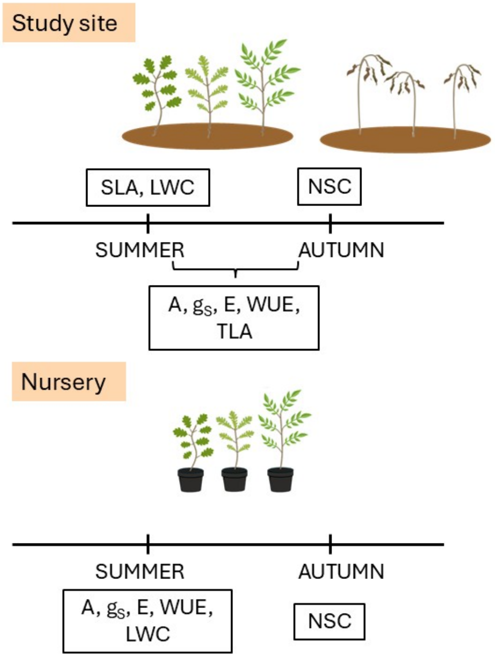

Figure S10. Experimental design. Leaf gas exchanges (i.e., A: net photosynthesis, g_S: stomatal conductance, E: transpiration rate, WUE: water use efficiency) were monitored in saplings of *Quercus pubescens* Willd., *Quercus cerris* L., and *Fraxinus ornus* L. (from left to right). Measurements were taken in summer on nursery saplings and in both summer and autumn on outplanted conspecifics, together with total leaf area (TLA). Specific leaf area (SLA) and leaf water content (LWC) were measured in summer. Non-structural carbohydrates (NSC) were quantified at the end of the growing season (autumn) in both alive and desiccated outplanted conspecifics at the study site, as well as in nursery saplings.

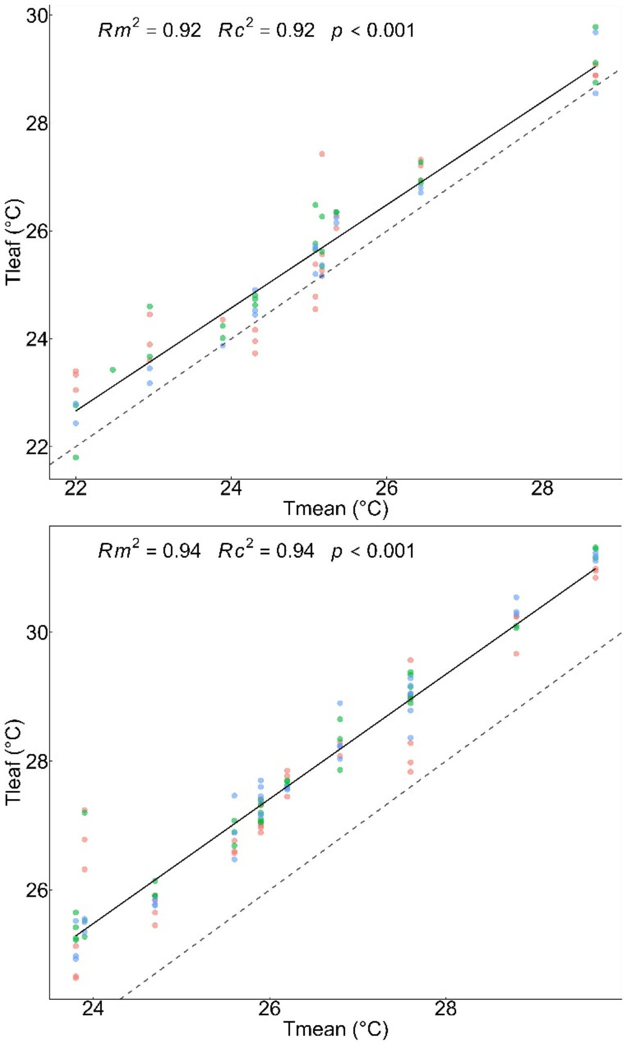

Figure S11. Relationship between leaf and air temperature at the nursery and study site. Linear mixed effects models between corrected leaf temperature and daily mean air temperature for the nursery (top panel) and study site (bottom panel), with sapling identity as a random effect. The points represent mean values per plant and sampling date. The colours indicate the species: *Fraxinus ornus* L. (red), *Quercus cerris* L. (green), and *Quercus pubescens* Willd. (blue). Marginal (R^2m) and conditional (R^2c) R^2 values and the model *p-value* (*p*) are reported at the top of each panel. The solid line represents the regression line, whereas the dashed line indicates the 1:1 line.

Supplementary Methods

Plant material. The plant material used for the reforestation was raised from seeds collected on the site of the following species: *Fraxinus ornus* L., *Quercus cerris* L., *Quercus pubescens* Willd., *Quercus ilex* L., and *Quercus suber* L. After germination, saplings were grown in 0.9 L plastic pots containing a peat–sand substrate (70:30 v/v) amended with slow-release Osmocote fertiliser for two years, then transferred to 2.6 L pots. Throughout the cultivation period in pots, they were irrigated under a shade house equipped with an overhead mist irrigation system at the "Regional Agency for the Development and Innovation of Agriculture of Latium" (ARSIAL) facilities. Saplings were outplanted in winter (January 2023) at three years of age, consistent with Italian guidelines for the use of native forest species (D.M. 17/05/2022), which specify that planting material should not exceed this age. ARSIAL planted 2,500 saplings in nine low-density tree clearings of the study area, using mulching discs around the basal stem to reduce weed competition and conserve soil moisture. Saplings were established in mixed-species groups with random spacing.

Table S1. Monitoring of nursery pot capacity. Pot weight (n = 3) during summer for each species, measured in the early morning after irrigation. The pot capacity was determined gravimetrically on the first date (June 12, 2023).

Date	Species	Mean pot weight at pot capacity (g)	Mean pot weight (g)
12/06/2023	*F. ornus*	1473.55 ± 24.14	1473.55 ± 24.14
16/06/2023	*F. ornus*	1473.55 ± 24.14	1511.813 ± 61.04
20/06/2023	*F. ornus*	1473.55 ± 24.14	1506 ± 56.48
23/06/2023	*F. ornus*	1473.55 ± 24.14	1518.94 ± 66.53
03/07/2023	*F. ornus*	1473.55 ± 24.14	1535.84 ±76.19
05/07/2023	*F. ornus*	1473.55 ± 24.14	1478.05 ± 79.49
12/06/2023	*Q. cerris*	1168.27 ± 118.10	1168.27 ± 118.10
16/06/2023	*Q. cerris*	1168.27 ± 118.10	1266.78 ± 102.36
20/06/2023	*Q. cerris*	1168.27 ± 118.10	1228.47 ±101.87
23/06/2023	*Q. cerris*	1168.27 ± 118.10	1240.67 ± 140.74
03/07/2023	*Q. cerris*	1168.27 ± 118.10	1167.35 ± 156.69
05/07/2023	*Q. cerris*	1168.27 ± 118.10	1076.42 ± 295.52
12/06/2023	*Q. pubescens*	1226.25 ± 112.52	1226.25 ± 112.52
16/06/2023	*Q. pubescens*	1226.25 ± 112.52	1280.30 ±160.86
20/06/2023	*Q. pubescens*	1226.25 ± 112.52	1311.09 ± 102.29
23/06/2023	*Q. pubescens*	1226.25 ± 112.52	1293.15 ± 115.03
03/07/2023	*Q. pubescens*	1226.25 ± 112.52	1290.54 ± 80.48
05/07/2023	*Q. pubescens*	1226.25 ± 112.52	1118.24 ± 121.42

Table S2. Estimation of leaf area from allometric data. Species-specific allometric models used to estimate leaf area (LA, cm^2) from leaf length (l, cm) and width (w, cm) applied to destructively sampled leaves (one leaf per plant, ten plants per species), for which LA, l and w were directly measured. The selected models based on the Akaike Information Criterion were then applied to additional leaves (three leaves per plant, ten plants per species) measured non-destructively, for which only l and w were recorded. Model fit is reported as R^2 and *p-value* (*p*), and predictive accuracy as mean absolute error (MAE) obtained by leave-one-out cross-validation.

**Species**	**Equation**	**R^2**	***p***	**MAE**
F. ornus	$LA = 2.42 - 2.31 \times l + 3.01 \times w + 0.42 \times l^2$	0.96	<0.001	0.62
Q. cerris	$LA = 8.94 - 3.32 \times l + 1.64 \times w + 0.48 \times l^2$	0.83	<0.001	1.61
Q. pubescens	$LA = -1.29 - 0.56 \times l + 2.55 \times w + 0.26 \times l^2$	0.97	<0.001	0.48
